# Novel molecular imaging approaches in oncology: towards a more accurate estimation of tumour response

**DOI:** 10.1097/CCO.0000000000001166

**Published:** 2025-07-09

**Authors:** Amy Rose Sharkey, Anum Pervez, Gary J.R. Cook

**Affiliations:** a Guy's and St. Thomas’ NHS Foundation Trust; b School of Biomedical Engineering and Imaging Sciences, King's College London; c King's College London & Guy's and St. Thomas’ PET Centre, St. Thomas’, Hospital, London, UK

**Keywords:** advanced imaging, molecular imaging, novel molecular tracers

## Abstract

**Purpose of review:**

With novel therapeutics improving cancer survival rates, the need for accurate treatment response assessment has become increasingly crucial. The Response Evaluation Criteria in Solid Tumours remains the standard imaging method for evaluation of treatment response, yet it has recognized limitations. Molecular imaging with targeted tracers offers earlier and more precise assessment of treatment efficacy, by capturing biological information beyond a change in tumour size. We discuss these recent advances, including tracers in clinical practice, and novel tracers in the pipeline, and how these can improve our assessment of treatment response.

**Recent findings:**

The development of novel tracers is enabling more precise cancer diagnosis, staging and treatment, and enables the use of targeted treatments. Upcoming tracers offer the potential to predict treatment response prior to treatment, eradicating the morbidity associated with ineffective therapy. Improved PET hardware, such as total body PET, allows accurate insights into factors such as tumour uptake kinetics, which can be paired with artificial intelligence tools to allow prediction of tumour characteristics.

**Summary:**

This review summarizes recent advances in molecular imaging, including tracers that target tumour metabolism, tumour microenvironment and other treatable tumour targets. These aim to improve treatment response assessment, with the hope of improving outcomes by offering personalized and timely treatment adjustments.

## INTRODUCTION

Accurate estimation of tumour response is becoming increasingly vital as novel therapeutics extend cancer survival rates. Assessment of treatment response has traditionally been assessed using the Response Evaluation Criteria in Solid Tumours (RECIST), with version 1.1 currently in use. This offers standardized objective criteria to compare baseline and posttreatment imaging to assess a patient's response to treatment. The delay between treatment initiation and posttreatment imaging leaves some patients undergoing treatment that is ineffective in treating their cancer. There is often morbidity associated with ineffective treatment, and inaccurate or slow assessment of treatment response introduces delays to starting alternative treatment strategies that may be more effective.

RECIST 1.1 defines complete response as the disappearance of all target lesions, partial response as at least 30% decrease in the sum of the diameters of the target lesions and progressive disease as at least 20% or more increase in the sum of the diameters of the target lesion. Nontarget lesions are not individually measured but should be described using standardized language. These criteria have limitations; bone metastases are assessable only if they have more than a 10 mm soft tissue component, which is rare, and sclerotic lesions are excluded. As a result, bone metastases – despite being a significant disease burden – are largely considered unmeasurable. A second issue is that lesion size alone is a crude indicator of response. A tumour may show response to treatment through necrosis, although overall lesion size is unchanged. A further issue is that some targeted treatments may not initially cause tumour shrinkage [1^▪▪^]. Immunotherapy can cause atypical imaging patterns of response, such as pseudoprogression, where tumour shrinkage or stabilization is observed after an initial increase in tumour size. While immune RECIST (iRECIST) captures pseudoprogression in immunotherapy patients, it struggles to assess atypical responses like dissociated response, where responding and nonresponding lesions co-exist. The presence of a dissociated response is seen with some systemic immunotherapy treatments and is often associated with a favourable prognosis. Failing to capture this is an issue, as the rate of dissociated response reported in different studies ranges from 3.3 to 47.8% [2], meaning iRECIST may often be failing to capture a frequently occurring atypical response, limiting the accuracy of iRECIST as a criterion for treatment response assessment.

The use of [^18^F]fluorodeoxyglucose ([^18^F]FDG) PET, as a marker of cancer glycolysis, has revolutionized the diagnosis, staging, and management of many cancers. Changes in [^18^F]FDG uptake tend to occur earlier than those seen with computed tomography (CT), improving the evaluation of response assessment [3,4]. However, [^18^F]FDG is not mechanistically specific enough to predict many cases of early treatment resistance. More targeted tracers, that specifically probe the molecular targets involved in drug efficacy and resistance, may be able to noninvasively detect and quantify the underlying molecular biological processes, with the ability to quantify biological intra-tumoral and inter-tumoral heterogeneity. These tracers have been developed across many different cancer processes, targeting tumour metabolism, microenvironment, proliferation, and hypoxia, and some target specific treatable receptors [5,6]. These provide valuable information about individual tumours, exposing heterogeneity and helping to determine the most suitable treatment. Furthermore, some of these novel tracers purport to predict a patient's response to treatment, including nonspecific systemic treatments, before the treatment is given, meaning that morbidity associated with ineffective treatment could be avoided. Some of these tracers can be labelled with α-emitting or β-emitting isotopes that can be used to treat cancer, via the combination of molecularly targeted imaging and therapy, with this combination of diagnostics and therapeutics known as theranostics [7].

This review aims to summarize recent updates in novel molecular imaging approaches in oncology, highlighting developing improvements in response assessment. 

**Box 1:**
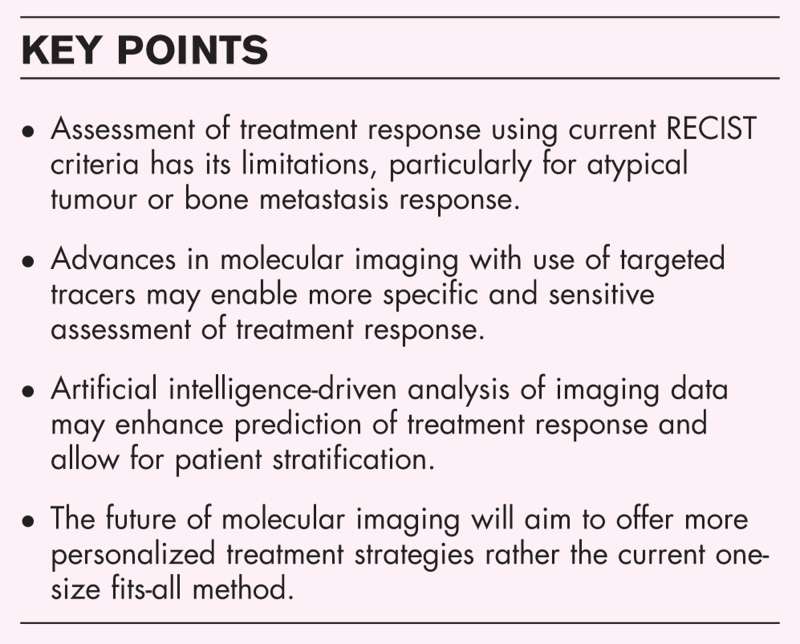
no caption available

## MOLECULAR TRACERS IN CLINICAL USE FOR RESPONSE ASSESSMENT

[^18^F]FDG PET/CT improves cancer response assessment by detecting tumoural metabolic changes earlier than tumour shrinkage. This is well documented in certain cancers [8], such as lymphoma, where it is commonly used with the Deauville criteria [9^▪^]. In other cancers, metabolic response is evaluated using either the European Organization for Research and Treatment of Cancer (EORTC) criteria [10] or the Positron Emission Tomography Response Criteria in Solid Tumours (PERCIST) [11]. Despite slight differences, studies show high levels of agreement between these two criteria [12]. However, they apply only to [^18^F]FDG avid lesions and lack validation for other tracers. Specific targeted molecular imaging probes, likely represent the future in assessment of cancer treatment response, and updated guidelines for standardization of response are required to take these into account.

In prostate cancer, prostate-specific membrane antigen (PSMA)-targeted PET/CT is used for diagnosis and increasingly for treatment response [13]. The Prostate cancer molecular imaging standardized evaluation (PROMISE) criteria provides a framework for classifying and quantifying PSMA tracer-avid disease [14]. While the prostate-specific antigen (PSA) blood test traditionally monitors treatment response, it has limitations, particularly in advanced metastatic prostatic cancer, which often displays intertumoural variability. PSMA PET/CT has recently proven superior to PSA monitoring in assessing treatment response [15^▪▪^].

**FIGURE 1:**
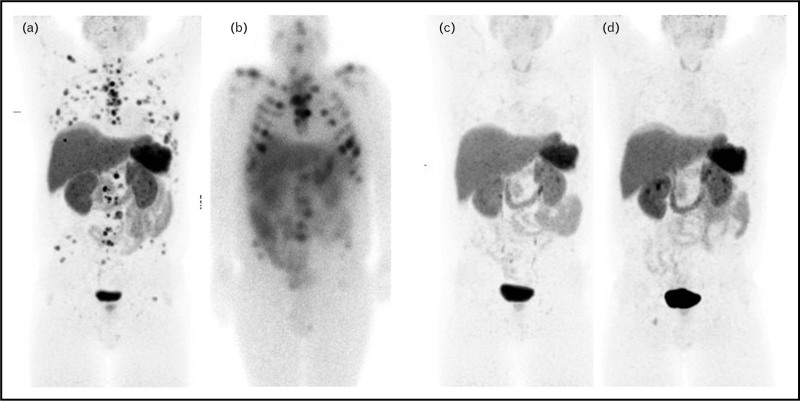
[^68^Ga]dotatate PET showing multiple dotatate-avid skeletal metastases (a). Post [^177^Lu]dotatate therapy SPECT (b), confirms therapeutic targeting. [^68^Ga]dotatate PET after completion of four cycles of [^177^Lu]dotatate therapy (c) shows complete metabolic response. [^68^Ga]dotatate PET (d), 6 months after therapy, confirms a maintained response.

Similarly, for neuroendocrine tumours, PET imaging with radio-labelled somatostatin receptor agonists, such as DOTATATE, can be used for both diagnosis and response assessment to peptide receptor radionuclide therapy (PRRT), including prediction of therapy outcome [16]. Example imaging is shown in Fig. 1.

## PROMISING TRACERS FOR RESPONSE ASSESSMENT

### [^18^F](4S)-4-(3-[18 F]fluoropropyl)-l-glutamate([^18^F]FSPG)

The degree of oxidative stress within cells can be imaged and quantified using the glutamic acid derivative tracer, [^18^F]FSPG, which is specifically transported by the cystine-glutamate antiporter system x_c_^-^. Animal cancer models have shown that changes in [^18^F]FSPG retention following effective therapy precede glycolytic changes, as indicated by [^18^F]FDG, and CT measured tumour volume [17,18], suggesting that [^18^F]FSPG could offer an early assessment of treatment response. Although [^18^F]FSPG has already been assessed as a diagnostic agent [19^▪^], no trials as of yet have been published looking at the value of [^18^F]FSPG in response assessment. One clinical trial which has completed, with the publication of results pending, is the evaluation of using [^18^F]FSPG in diagnosis, prediction, and evaluation of treatment response in a variety of metastatic cancer types (NCT02599194), and another is in progress in head and neck squamous cell cancer (HNSCC) and nonsmall cell lung cancer (NSCLC) (NCT05889312).

### [^18^F]-3’-deoxy-3’-fluorothymidine ([^18^F]FLT)

The thymidine analogue [^18^F]FLT can be used as a nonspecific marker of tumour proliferation [20,21]. Results with [^18^F]FLT PET in the assessment of treatment response have been mixed; when compared with CT in a cohort of patients with mesothelioma and NSCLC, a decrease in proliferation as measured by the maximum standardized uptake value (SUV_max_), a semi-quantitative measure of lesion activity, did not precede a decrease in size as measured by CT [22]. However, when compared with [^18^F]FDG PET in patients with NSCLC undergoing radical chemoradiation therapy, [^18^F]FLT was a more sensitive tracer of early treatment response [23].

### Fibroblast activation protein inhibitor

Fibroblast activation protein (FAP) is highly expressed in the stroma surrounding tumours and is a specific surface marker for cancer-associated fibroblasts, which are the target of FAP inhibitor (FAPI) PET. FAPI PET is useful in many different tumour types, and disease extent on [^68^Ga]FAPI-46 PET/CT is a predictor of short overall survival in various solid tumours [24^▪^]. Early studies looking at response assessment with FAPI PET are promising. A small study of patients with invasive lobular breast cancer (a cancer in which [^18^F]FDG performs poorly) found a strong correlation between [^68^Ga]FAPI tumour volume posttreatment and blood biomarkers in six patients before and after treatment (hormonal treatment in three patients, chemotherapy in two patients and antibody–drug conjugates in one patient) [25]. There are also early studies assessing the use of FAPI PET in the evaluation of neoadjuvant chemotherapy (NAC) response; a study of 22 patients with newly diagnosed breast cancer found changes in [^68^Ga]FAPI uptake after two cycles of NAC were predictive of pathological complete response [26^▪^].

### ^89^Zirconium [^89^Zr] trastuzumab (HER2) PET/CT

Intra-patient HER2 heterogeneity expression in breast cancer [27] has spurred interest in [^89^Zr]trastuzumab PET/CT for whole-body HER2 assessment, aiding treatments like HER2-targeting trastuzumab emtansine (T-DM1), which targets tumour HER2 status. The ZEPHIR trial, in which patients with advanced HER2 positive breast cancer underwent HER2 PET/CT and [^18^F]FDG PET/CT before T-DM1 initiation, found 93/265 (35%) lesions were HER2-negative, and of these, 18 (19%) lesions (from 11 patients), responded anatomically after three T-DM1 cycles, resulting in an 81% NPV of the HER2 PET/CT. When combined with early metabolic response assessment using [^18^F]FDG PET/CT, performed before the second T-DM1 cycle, NPVs of 91 and 100% were reached in predicting lesion-based and patient-based (RECIST1.1) response, respectively [28^▪^]. HER2 PET/CT, alone or in combination with early [^18^F]FDG PET/CT, may, therefore, be able to identify breast cancer lesions with a low probability of clinical benefit from T-DM1, allowing stratification of patients.

### 16a-^18^F-fluoro-17b-estradiol ([^18^F]FES)

[^18^F]FES is a tracer, which quantifies in-vivo oestrogen receptor expression, meaning [^18^F]FES PET/CT can noninvasively assess the oestrogen receptor status of multiple tumour lesions in one scan. [^18^F]FES, alongside [^18^F]FDG PET/CT, has been shown to alter patient management by providing additional diagnostic insights obtained in a scan, with one study demonstrating that [^18^F]FES changed management in 5 of 19 (26.3%) breast cancer patients [29]. [^18^F]FES PET/CT might also predict treatment efficacy; a phase II trial evaluating the efficacy and safety of adding fulvestrant, an oestrogen receptor antagonist, to NAC in oestrogen receptor-positive/HER2-negative patients with locally advanced breast cancer, found that the SUV_max_, SUV_mean_, and total lesion-oestrogen receptor expression of [^18^F]FES PET/CT in sensitive patients were significantly higher than those in nonsensitive patients (*P* < 0.05). Furthermore, there was significant correlation of these parameters with the histopathological grade, and the pretreatment and posttreatment change in oestrogen receptor expression (*P* ≪ 0.05) [30^▪▪^], suggesting [^18^F]FES PET/CT could be used to stratify patients pretreatment.

### Tracers targeting programmed death-ligand 1

**FIGURE 2:**
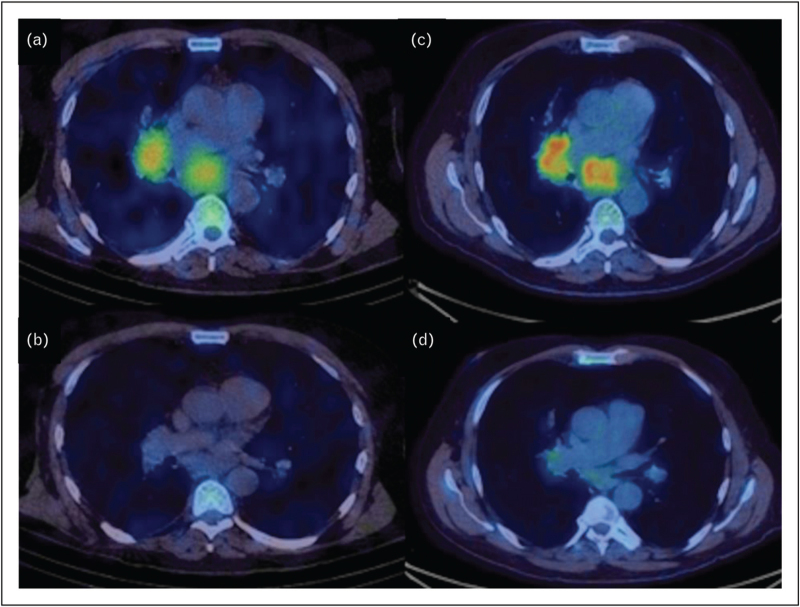
[^99m^Tc]NM01 (a PD-L1 targeting nanobody) showing high PD-L1 expression in non-small cell lung cancer right hilar and subcarinal nodes before treatment with pembrolizumab (a), and [^99m^Tc]NM01 scan at 9 weeks showing reduction in PD-L1-expressing tumour (b). This is compared with [^18^F]FDG PET demonstrating high tumour uptake (c) with a significant 9-week metabolic response (d) as predicted by high PD-L1 baseline expression.

Development of immunotherapy has revolutionized lung cancer outcomes, doubling the median overall survival in metastatic NSCLC patients with high programmed death-ligand (PD-L1)-expressing tumours [31]. The PD-L1 targeting tracer, [^68^Ga]NOTA-WL12, has been trialled in patients with advanced NSCLC, finding a positive correlation between tumour uptake (SUV_peak_) and PD-L1 immunohistochemistry results, suggesting PD-L1 PET before therapy may predict the therapeutic efficacy of targeted immunotherapy [32]. Furthermore, a recent study has demonstrated intertumoural and temporal heterogeneity of PD-L1, as measured by [^99m^Tc]NM-01 SPECT/CT, in patients with advanced NSCLC receiving anti-PD-1 therapy, and found that the baseline [^99m^Tc]NM-01 tumour to blood pool ratio was predictive of early metabolic [^18^F]FDG-PET/CT response to anti-PD-1 therapy [33] (PD-L1 imaging highlighted in Fig. 2). This is in keeping with findings using the alternative anti-PD-L1 tracer, ^89^Zr-atezolizumab, which showed clinical responses were better correlated with pretreatment PET signal than with immunohistochemistry, or RNA-sequencing-based predictive biomarkers, suggesting molecular PET imaging assessment of PD-L1 status can predict clinical response [34].

### Tracers targeting TROP2 expression

TROP2 is overexpressed in different tumour tissues and plays roles in cell proliferation, invasion, migration, apoptosis, and treatment resistance [35], and TROP2 PET imaging can noninvasively assess tumoural TROP2 expression. A small clinical study using [^68^Ga]-MY6349 PET/CT assessed TROP2 expression in various cancers. Results showed a strong correlation, as determined by immunohistochemistry, between TROP2 levels and [^68^Ga]-MY6349 uptake at primary and metastatic tumour sites. Compared to [^18^F]FDG PET/CT, [^68^Ga]-MY6349 PET/CT showed higher tumour uptake in certain cancers, including breast, prostate, and thyroid cancers. Against [^68^Ga]-PSMA-11, [^68^Ga]-MY6349 PET/CT demonstrated comparable lesion uptake, but better tumour-to-background contrast in primary and metastatic prostate cancer, allowing visualization of more metastatic lesions. This suggest TROP2 PET could be used to identify patients suitable for TROP2-targeted drug treatment.

## THERANOSTICS

Molecular imaging also has a more direct therapeutic potential, as the targets traditionally used for PET and SPECT imaging can be used in a treatment context by labelling with either β-emitting or α-emitting radionuclides, thus combining therapy and diagnosis. This concept is referred to as theranostics. It has been used for many decades in nuclear medicine, starting with the use of radioiodine pharmaceuticals for thyroid cancer in 1946 [36].

Use of theranostics has increased, with targeted treatment now including radium-223 for treatment of skeletal metastatic disease [37], the use of [^177^Lu]-dotatate therapy for neuroendocrine tumours [38], and [^177^Lu]-PSMA-617 therapy for prostate cancer [5]. Promising new theranostic targets are emerging, one of which is B7-H3, an immune checkpoint molecule and a costimulatory/coinhibitory immunoregulatory protein, which is overexpressed in tumour tissues in various solid malignancies, and increased expression is associated with advanced disease and worse patient survival [39^▪^]. Preclinical studies of anti-B7-H3 agents, including those using a conjugated antibody radiolabelled with ^89^Zr as a PET tracer to detect B7-H3 expression, have exhibited encouraging results [40^▪^], suggesting anti-B7-H3 agents could have potential as a clinical theranostic agent.

## ADVANCES IN PET

One potential concern with the use of serial PET/CT for assessment of treatment response is the associated radiation dose. However, advances in the field of PET imaging have now made this a near negligible concern. The development of long axial field of view PET, has represented a step change in oncological imaging. It has several major benefits, including a gain in sensitivity [41], which can be used to reduce administered activity [42] and scan acquisition times [43]. There are also improved capabilities in dynamic imaging, facilitated by long axial field of view PET [44^▪^].

## ARTIFICIAL INTELLIGENCE AND RADIOMICS

Several different artificial intelligence methodologies have been used in the assessment of treatment response and prediction of treatment response with molecular imaging. PET imaging generates a huge amount of data, and AI could be used to assess imaging features to delineate, which are the most predictive parameters in different cancer types. This approach has been evaluated in prostate cancer, where an AI model integrating PSMA PET/CT results with standardized imaging criteria successfully tracking individual tumour changes, indicating its potential as a novel assessment tool for treatment response [45^▪^].

Radiomics may be able to assess the heterogeneity of uptake information of [^18^F]FDG and other molecular imaging probes, which could then be exploited to predict or measure response more accurately than standard metrics [46]. PET/CT-based radiomic features have demonstrated strong potential in predicting PD-L1 expression status and thus could be used to preselect patients who may benefit from PD-1/PD-L1-based immunotherapy, for example [47]. Radiomic signatures derived from [^18^F]FDG PET images have been shown to effectively distinguish various breast cancer characteristics. It has been shown that extracting metabolic and radiomic texture features from [^18^F]FDG PET/CT, and combining these features with clinical parameters, can predict pathological complete response (pCR) to NAC in patients with HER2 and triple negative breast cancer [48^▪^]. [^18^F]FDG PET/MR) may offer additional benefits. In a study of 73 patients with newly diagnosed treatment-naive breast cancer, multiparametric [^18^F]FDG PET/MRI-based radiomic analysis was able to predict pCR with a NPV of 79.5% [49].

## CONCLUSION

At present, there are limited options with imaging to predict treatment response, and there is a potential delay between starting a cancer treatment and the assessment of whether or not that treatment is working when using conventional size-based criteria. Advances in molecular imaging, in terms of novel tracers that probe therapeutic tumour targets or mechanisms of resistance, together with improved scanning capabilities, offer the promise of early and accurate treatment prediction and response assessment, with the added benefits of being noninvasive and offering whole body coverage.

## Acknowledgements

*None*.

### Financial support and sponsorship

*This work was supported by the Medical Research Council (MR/Y008987/1), the Cancer Research UK National Cancer Imaging Translational Accelerator (C1519/A28682), Breast Cancer Now (2018JulPR1092), Rosetree's Trust (PGL23/100049), Guy's Cancer Charity (CC220204), and the Royal College of Radiologists.*

### Conflicts of interest

*There are no conflicts of interest.*

## REFERENCES AND RECOMMENDED READING

Papers of particular interest, published within the annual period of review, have been highlighted as:

▪ of special interest
▪▪ of outstanding interest
